# Cross-scale habitat structure driven by coral species composition on tropical reefs

**DOI:** 10.1038/s41598-017-08109-4

**Published:** 2017-08-08

**Authors:** Laura E. Richardson, Nicholas A. J. Graham, Andrew S. Hoey

**Affiliations:** 1 ARC Centre of Excellence for Coral Reef Studies, James Cook University, Townsville, QLD 4811 Australia; 2 Lancaster Environment Centre, Lancaster University, Lancaster, LA1 4YQ United Kingdom

## Abstract

The availability of habitat structure across spatial scales can determine ecological organization and resilience. However, anthropogenic disturbances are altering the abundance and composition of habitat-forming organisms. How such shifts in the composition of these organisms alter the physical structure of habitats across ecologically important scales remains unclear. At a time of unprecedented coral loss and homogenization of coral assemblages globally, we investigate the inherent structural complexity of taxonomically distinct reefs, across five ecologically relevant scales of measurement (4–64 cm). We show that structural complexity was influenced by coral species composition, and was not a simple function of coral cover on the studied reefs. However, inter-habitat variation in structural complexity changed with scale. Importantly, the scales at which habitat structure was available also varied among habitats. Complexity at the smallest, most vulnerable scale (4 cm) varied the most among habitats, which could have inferences for as much as half of all reef fishes which are small-bodied and refuge dependent for much of their lives. As disturbances continue and species shifts persist, the future of these ecosystems may rely on a greater concern for the composition of habitat-building species and prioritization of particular configurations for protection of maximal cross-scale habitat structural complexity.

## Introduction

The physical structure of habitats is integral to the organization, function, and resilience of ecosystems^1–3^, and therefore the provision of ecosystem goods and services. The diversity and abundance of taxa such as birds, small mammals, lizards, and fish, commonly correlate with the structural complexity of habitats across a range of ecosystems^4^. Specifically, the availability of microhabitats over a range of spatial scales provides associated organisms of different sizes with refuge from predation, allows for greater niche differentiation and can facilitate other species by mediating competition, and reducing environmental conditions to tolerable levels^5^. Animals often use their environment at spatial scales relative to their body-size, for example spatial refugia from predators^3^. However, habitat structural complexity at one scale of measurement is not necessarily synonymous with complexity at other scales (e.g. ref. 6). The availability of fine and coarse scale structural complexity often varies among habitats, with direct implications for the distribution of organisms^7, 8^, the maintenance of ecosystem processes^9, 10^, and the resilience of communities^2^.

The structural complexity of habitats is typically created by communities of living organisms (i.e. habitat-forming organisms such as trees, canopy-forming seaweeds, oysters, wetland grasses, and corals), as well as abiotic features such as the underlying geomorphology, and/or three-dimensional structures of dead organisms^6, 11^. Importantly, both the abundance and species composition of habitat-forming organisms can have a strong influence on the structural complexity of habitats. For example, the habitat structural complexity of forests varies with tree species composition^12, 13^; wetland habitats vary with the composition of forbs, grasses and rushes^14^; and the structure of subtidal temperate reefs is dependent on the species composition of canopy-forming seaweeds^15^. Similarly on coral reefs, habitat structural complexity is likely underpinned by the relative abundance of component coral species^16^, and can vary independently of total coral cover^17^. Corals are structurally diverse taxa, characterized by a range of morphologies (e.g. branching, foliose, massive, or tabulate) that are determined by evolved life history strategies^18^, genetic variation, and environmental phenotypic plasticity^19^. Even within these morphological groupings there is considerable variation among species in the size and shape of morphological features (e.g., length and spacing between branches, branching pattern) and hence the interstitial spaces created within, underneath and between colonies^20–22^.

Globally, pervasive anthropogenic disturbances are reducing species populations, leading to biotic homogenization of communities and changes to the functioning of ecosystems^23, 24^. On tropical reefs, climate-change induced warm-water anomalies, severe storms, land-based sources of pollution and sedimentation, overfishing, and predation by crown-of-thorns starfish are leading to marked declines in the abundance, and changes in the composition of habitat-forming corals^25, 26^. Differential susceptibilities to disturbance and variation in life-histories among coral species are causing non-random homogenization of coral assemblages, often dominated by species that are relatively more tolerant to stress, or fast growing and quick to colonize^18, 27–29^. Some of the most structurally complex corals, such as taxa with branching morphologies, are the most susceptible to a range of disturbances, including storms^30^, thermal-bleaching^31^, and crown-of-thorns starfish^32^. While reductions in the abundance of these structurally complex corals is typically related to reductions in the structural complexity of habitats^33^, disturbances can also lead to increases in habitat structural complexity, particularly where reefs persist as altered coral-dominated systems^34^. Consequently, changes in coral composition will likely impact the habitat structural complexity of coral reefs^34^, with direct implications for the capacity of those reefs to maintain reef functions^35, 36^, and the provision of coral reef ecosystem goods and services^37, 38^.

**Figure 1:**
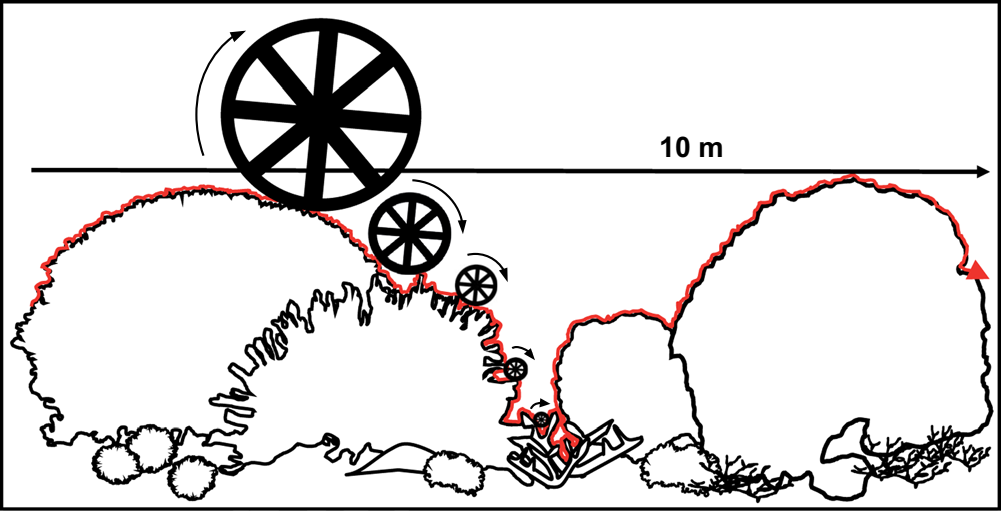
Contour distance estimated with five wheels of different diameters (4–64 cm) along a 10 m transect. Wheels closely follow the surface structure of the benthos and number of rotations are counted. Small wheels fit into more holes than larger wheels and thus estimate greater contour distance.

Shifts in the composition of habitat-building coral species are predicted to persist into the future^26, 39^. Therefore, identifying the structural characteristics of particular coral configurations is critical for the conservation of those systems. However, an understanding of the inherent variation in cross-scale structural complexity of coral reefs is currently lacking. To this end, this study aimed to investigate the influence of coral species composition on cross-scale patterns of habitat structural complexity, at spatial scales of measurement relevant to fish refuge selection (adapted from ref. 8). Cross-scale structural complexity was quantified at randomly selected sites at Lizard Island in the northern Great Barrier Reef, Australia (14°41′S, 145°27′E), following a step-length geometric series using contour distance measuring wheels of different diameters (4–64 cm) along 10-m transects at each site (Fig. 1; see Supplementary Table S1). Specifically, we assessed i) the cross-scale structural complexity of four coral habitats with distinct species configurations and degraded (<10% total coral cover) reef habitat, and ii) cross-scale colony level structural complexity of the dominant coral species at our study location to elucidate the relationship between the complexity of taxa-specific morphologies and colony size.

## Results

### Habitat classification

**Figure 2:**
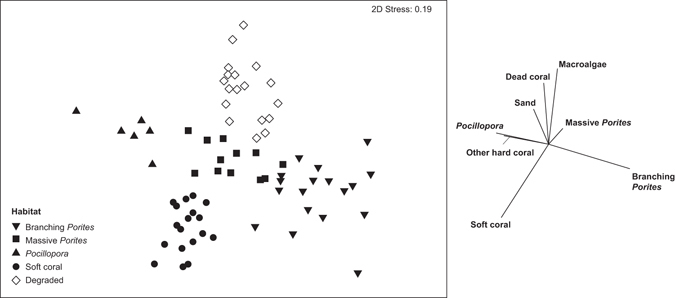
Non-metric multidimensional scaling analysis showing variation in benthic composition among surveyed reef habitats at Lizard Island, using transect level square root transformed data. The relative contribution of benthic categories to the observed variation in benthic composition are illustrated (>0.2 Pearson correlation).

Benthic composition varied among the twelve sites, with five distinct habitat groups identified by MDS and hierarchical clustering of benthic composition (Fig. 2). PERMANOVA supported these groupings with significant differences in benthic composition among the five groups (Pseudo-*F* = 11.22, *df* = 4, *P* = 0.0001; all pairwise comparisons *P* = 0.0001; see Supplementary Table S2). SIMPER analysis indicated dominant taxa and substrate types (i.e., *Porites cylindrica*, massive *Porites* – mostly *P. lutea*, *Pocillopora damicornis*, soft coral, dead coral and macroalgae) consistently contributed to average similarity within, or dissimilarity between groups (see Supplementary Table S2). Cover of these dominant coral taxa (including soft coral) ranged from 51.5–90.1% of total live coral in coral-dominated sites (mean total coral cover 51.3% ± 4.6 SE). Conversely, the grouping characterized by dead coral and pavement, rubble, and macroalgae (79.4% ± 1.2 SE benthic cover), had significantly less live coral cover (10.5% ± 1.8 SE; lme, *F*_4, 7 = _25.83, *P* = 0.0003; Tukey, all *P* ≤ 0.03). Among the coral-dominated groupings, total live coral cover was higher at sites dominated by *Porites cylindrica* than those characterized by *P. lutea*, *Pocillopora damicornis*, or soft coral which had comparable cover (lme, *F*_4,7 = _25.83, *P* = 0.0003). Sites were classified by habitat groupings according to dominant substrata as follows: *Porites cylindrica* (hereafter ‘branching *Porites*’; 3 sites), *P. lutea* (hereafter ‘massive *Porites*’; 2 sites), *Pocillopora damicornis* (hereafter ‘*Pocillopora*’; 1 site), soft coral (3 sites), and degraded (3 sites) for all subsequent analyses.

### Habitat structural complexity

**Figure 3:**
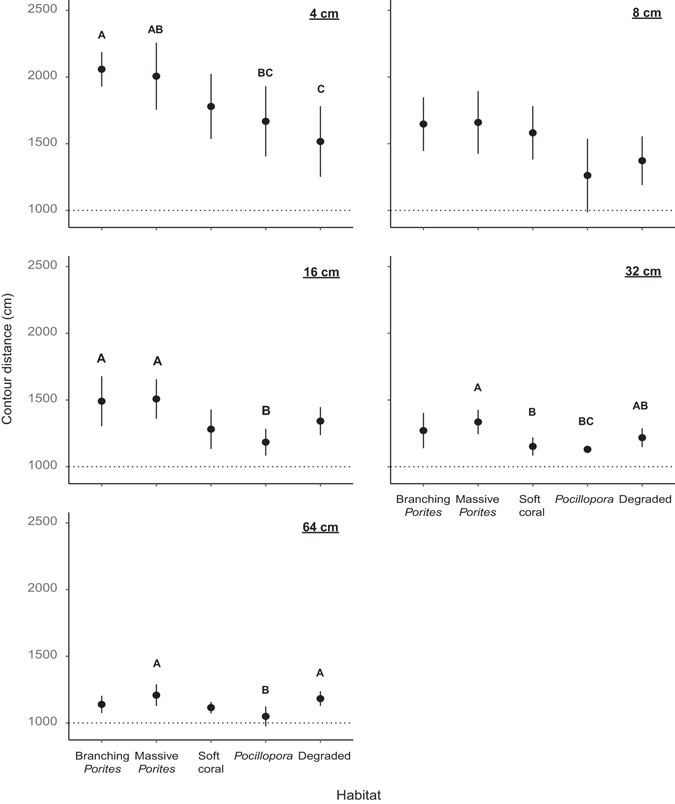
Modelled contour distance (±95% confidence intervals) measured along 10 m transects at scales 4–64 cm with measuring wheels (wheel diameters, cm: 3.99; 7.97; 15.95; 31.89; 63.79), in different coral reef habitats (*n* = 4–12 per habitat). Significant differences between habitats revealed by *post hoc* Tukey pair-wise comparisons are illustrated by the pairing of letters (P < 0.05).

The structural complexity of habitats quantified using distance measuring wheels of different diameters (4–64 cm) that followed the reef surface contour along four 10-m transects at each site changed with scale of measurement, and varied among habitats with similar levels of coral cover (Fig. 3; see Supplementary Tables S1 and S3). Modelling multi-scale contour distance with coral cover and benthic composition indicated that at the smallest scales (4–8 cm), total coral cover was a significant predictor of contour distance, but variation in benthic composition (habitat type) was also in the top models with a relative importance of 0.27 (4 cm scale) and 0.17 (8 cm scale). Total coral cover was not present in the top models for structural complexity at larger scales (16–64 cm), indicating that benthic composition better predicted variation in contour distance measured among sites (Table 1). Null models featured in the top models for structural complexity at the 8, 16, and 64 cm scales, indicating high variability among transects (scales 8 and 16 cm) and/or sites (64 cm) (see extracted variance components in Supplementary Table S1). Broadly, structural complexity varied significantly among habitats at all scales except 8 cm, though inter-habitat differences were not consistent among scales. Branching *Porites* and massive *Porites* habitats were generally more complex than soft coral, *Pocillopora* and degraded habitats at the small and intermediate scales (4–16 cm). The structural complexity of branching *Porites* habitats reduced to intermediate levels at the 32 cm scale, comparable with degraded habitats; and massive *Porites* and degraded habitats were most complex at the largest scale (64 cm) (Fig. 3; see Supplementary Table S3).

**Table 1:** Top candidate models selected to describe the relationship between habitat structural complexity across scales (4–64 cm), with total coral cover and habitat type (benthic composition).

Scale (cm)	Model rank	AICc	*df*	logLik	ΔAICc	wAICc	Total coral cover (%)	Habitat	Model output (lme)
4	1	665.26	8	−322.68	0.00	0.73	**X**		*F*_1,33_ = 23.06, ***P*** ** < 0.0001**
	2	667.29	11	−318.76	2.03	0.27		**X**	*F*_4,7_ = 6.73, ***P*** ** = 0.02**
8	1	668.63	8	−324.37	0.00	0.57	**X**		*F*_1,33_ = 5.03, ***P*** ** = 0.03**
	2	670.16	7	−326.60	1.53	0.26	Null model		*F*_1,34_ = 1112.41, ***P*** ** < 0.0001**
	3	671.05	11	−320.64	2.42	0.17		**X**	*F*_4,7_ = 3.32, ***P*** ** = 0.08**
16	1	634.33	11	−302.28	0.00	0.80		**X**	*F*_4,7_ = 6.18, ***P*** ** = 0.02**
	2	637.14	7	−310.09	2.80	0.20	Null model		*F*_1,34_ = 1187.59, ***P*** < **0.0001**
32	1	NA	11	−255.26	NA	NA		**X**	*F*_4,7_ = 8.81, ***P*** ** = 0.01**
64	1	517.18	11	−243.71	0.00	0.69		**X**	*F*_4,7_ = 4.34, ***P*** ** = 0.04**
	2	518.80	7	−250.93	1.62	0.31	Null model		*F*_1,34_ = 4792.91, ***P*** < **0.0001**

Models are ranked by Akaike’s information criteria (AICc), with all models within ΔAICc < 3 of the top ranked model. The relative weight of evidence for each model is indicated by Akaike weight (wAICc), and the variables present in each model are indicated with an X. Null models refer to variance explained by site or transect level sampling Outputs are presented for each model, tested using Site as a random effect, and fitted with a constant variance structure to allow for heterogeneity at all scales.

**Figure 4:**
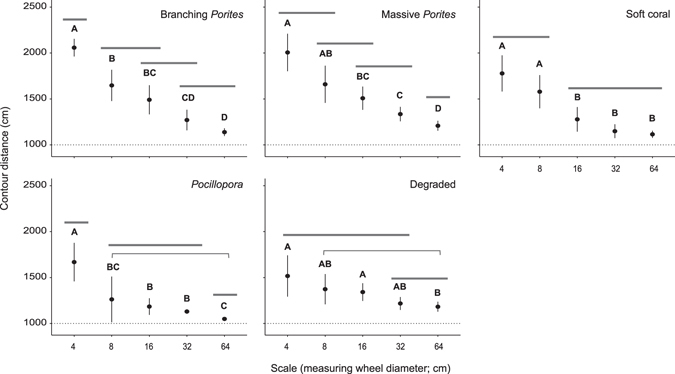
Modelled contour distance (±95% confidence intervals) measured using wheels representing scales 4–64 cm (wheel diameters, cm: 3.99; 7.97; 15.95; 31.89; 63.79), within each habitat. Significant differences between habitats revealed by linear mixed effects modelling and *post hoc* Tukey pair-wise comparisons are illustrated by the pairing of letters (P < 0.05). Grey bars across scales further illustrate similarities in structural complexity across scales of measurement in each habitat. Thin grey bars in *Pocillopora* and Degraded habitats denote similarities in contour distance over scales at either end of the bar (i.e. non-consecutive sales).

Contour distance travelled along transects declined with increasing wheel size in all habitats, however, the magnitude of change and cross-scale patterns of structural complexity varied among habitats (Fig. 4). Branching *Porites* and massive *Porites* had the greatest variation in complexity across scales, while degraded habitat had the least (Fig. 4; see Supplementary Table S4). Habitat structural complexity was identified at four distinct scales in the massive *Porites* and branching *Porites* habitats, three in *Pocillopora*, and at two distinct scales in the soft coral and degraded habitats. In soft coral, massive- and branching-*Porites* habitats, structural complexity at the two smallest scales (4 and 8 cm) was significantly greater than structural complexity at the two largest scales (32 and 64 cm). However, in the *Pocillopora* and degraded habitats these distinctions between scales were less apparent. Within the *Pocillopora* habitat, structural complexity at the smallest scale (4 cm) was greater than at the remaining scales (8–64 cm), while in the degraded habitat structural complexity was similar across all but the largest scale (64 cm).

### Colony level structural complexity

**Figure 5:**
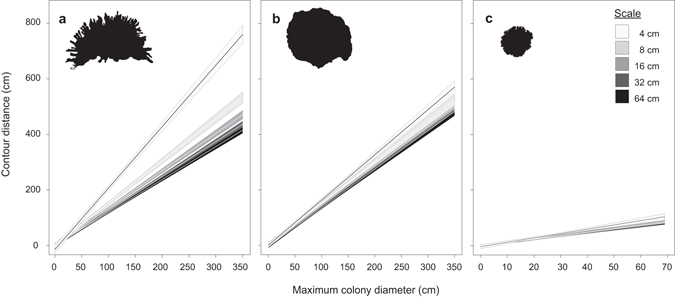
Relationships between maximum colony diameter (cm) and contour distance travelled by measuring wheels of diameters 4–64 cm (±95% confidence intervals): (**a**) *Porites cylindrica* (R^2^ = 0.93–0.97); (**b**) massive *Porites* (R^2^ = 0.95–0.97); and (**c**) *Pocillopora damicornis* (R^2^ = 0.68–0.91).

Colony level analyses revealed strong linear relationships between the structural complexity of massive *Porites*, *P. cylindrica* and *Pocillopora damicornis* and colony size for each taxa, with constant relationships between contour distance and colony diameter at all five scales (correlation coefficients ranged from 0.96–0.97, 0.93–0.97, and 0.68–0.91, respectively) (Fig. 5, see Supplementary Table S5). Visual inspection of regression slopes suggests that both *Porites* taxa were more structurally complex than *Pocillopora damicornis* across their size ranges at all scales. *Porites cyclindrica* colonies appear more structurally complex than massive *Porites* colonies of the same size at the 4 cm scale, and to a lesser degree at the 8 cm scale. Conversely, massive *Porites* colonies appear more complex at the 16–64 cm scales (Fig. 5, see Supplementary Table S5).

## Discussion

Disturbance induced biotic homogenization threatens the architecture of habitats at ecologically relevant scales and the resilience of ecosystems^12, 24^. Previous studies have described the physical flattening of habitats associated with the loss of key organisms such as trees, kelp, and corals, and the often profound consequences for biodiversity and related ecosystem services^12, 33, 40^. Here, we show that the habitat structural complexity of the studied reefs was inextricably tied to the identity of constituent habitat-building species, and was not shaped solely by the absolute cover of corals. Importantly, the structural complexity of habitats changed with scale of measurement. The greatest differentiation in habitat structural complexity was at the smallest (4 cm) and most vulnerable scale of measurement^41^. Furthermore, cross-scale structural complexity varied among all habitats, evident at four distinct scales in both *Porites* habitats, three in *Pocillopora* habitat, and two in soft coral and degraded habitats. It should be noted that the less rigid biota such as soft corals and large macroalgae likely provide elements of structural complexity that may not be effectively captured by the methods used in this study. These findings have substantial implications for the relative suitability of coral reef habitats for associated organisms that are refuge dependent, including other reef invertebrates and small-bodied reef fishes^41, 42^.

Differential habitat structural complexity across scales emphasizes the role of species composition in shaping the physical architecture of reef ecosystems. For example, we show that at small to mid-scales (4–16 cm), habitat structural complexity was greatest in massive and branching *Porites* habitats, relative to *Pocillopora*, soft coral and degraded habitats, while at larger scales (32–64 cm), the greatest structural complexity was in massive *Porites* and degraded habitats. Similar differences were evident in colony level complexity for both *Porites* taxa versus *Pocillopora*. Notably, the greatest differentiation in habitat structural complexity was evident at the smallest scales of measurement (4–8 cm). Structural complexity at these scales is largely determined by variation in the surface morphology of individual coral colonies (see Fig. 5), and likely provides the most benefit to small bodied and/or juvenile fishes subject to high risk of predation^41^. Branching *Porites* colonies were notably more structurally complex at the 4 cm scale, relative to massive *Porites* colonies of the same size, though relatively high contour distances were also observed in the latter (Fig. 5; Supplementary Table S5). Branching *Porites* species such as *P. cylindrica* create intricate and discrete interstitial spaces between their branches, whilst large colonies of massive *Porites* species can form fine scale corrugations or crevices in their otherwise relatively planar surfaces thereby providing small-scale microhabitats for small-bodied reef organisms^43, 44^. Despite greater complexity of branching *Porites* colonies, the differentiation in complexity between *Porites* taxa was lost at the transect level possibly due to variable size distributions of colonies in these habitats. Overall, the high contour distances measured in these *Porites* habitats likely reflect the typically large size of the branching and massive *Porites* colonies, as well as the undercut areas beneath and the vertical relief between colonies.

Whilst *Pocillopora* habitat was the least structurally complex of the four coral-dominated habitats across all scales, at scales finer than those considered in this study (i.e. <4 cm), *P. damicornis* is structurally intricate, and likely provides important refugia for many reef fishes^45^. However, due to its small size, brooding reproduction and fast growth rates^46^, *Pocillopora* dominated reefs can be characterised by multiple, tightly-aggregated colonies of similar sizes, offering little relief between them that might otherwise provide greater structural relief across all scales, but particularly at an inter-colony scale of approximately 8–32 cm.

The role of live coral in providing structurally complex tropical reef habitats has received much attention^33, 47^. However, the absolute cover of live corals alone does not capture all of the inherent variation in habitat structural complexity^17, 34, 48, 49^. We found that total coral cover was a good predictor of structural complexity at the two smallest scales (4–8 cm), but the inclusion of habitat composition further increased the predictive capacity of the models. At the larger scales (16–64 cm), the relationship between total coral cover and structural complexity of the habitat broke down. Our findings are consistent with studies from the Caribbean showing that whilst the fine-scale habitat structural complexity of reefs (0.7 cm scale) increases with coral cover, much of the variance in complexity at high levels of coral cover results from the dominance of particular corals^16^. It is important to note that despite differences in structural complexity among habitats at the larger scales (i.e. 8–64 cm), variation in the underlying reef structure, as well as the likely contribution of other benthic organisms, introduced substantial variation in complexity at the transect and site sampling levels. The contribution of the underlying substrata (geomorphological structure and dead reef matrix) to the structural complexity of reef habitats was further highlighted by the greater structural complexity of the degraded habitat found at larger scales (32–64 cm). This supports previous findings comparing multiscale complexity of coral-, and macroalgal-dominated habitats^8^.

The availability of habitat structural complexity across a range of scales is important for maintaining the organisation of associated organisms, including body-size distributions, food web structure and ecosystem functioning^2, 50^. We found that cross-scale habitat structural complexity varied with coral composition, with multi-scale structure most distinguished in branching and massive *Porites* habitats, relative to *Pocillopora* and soft coral habitats. These coral-dominated habitats all contrasted with the low relief degraded reef habitats across all scales. As reef habitats degrade, they become flatter and more structurally homogenous^33^, providing fewer potential refuges at different scales^8^, though large stands of macroalgae also contribute to elements of structural complexity^51^. Broadly, the structural complexity of coral reef habitats is evident both within and between colonies, and at larger scales that capture the corrugations of the underlying substratum^6^. More specifically however, our results reveal that cross-scale habitat structural complexity is influenced by the composition of coral species, with habitats providing structure ranging from just two scales measured in this study (e.g. soft coral and degraded habitats), to four scales (e.g. massive-, and branching *Porites* habitats). Interestingly, whilst the *Porites* habitats both displayed structural complexity at four distinct scales of measurement, cross-scale complexity varied between them. For example, structural complexity at the 4 cm scale differed to complexity at the 8 cm scale in branching *Porites* habitats, but not in massive *Porites* habitats. This was supported by colony level analyses indicating greater complexity of branching *Porites* at the 4 cm scale relative to massive *Porites* resulting from the interstitial spaces created between branches of *P. cylindrica*. Similarly, structural complexity at the 64 cm scale was distinct from complexity at the smaller scales in massive *Porites* habitats, but not branching *Porites* habitats, likely due to the overhangs often created by large colonies of massive *Porites*. The only shared variation in structural complexity occurred between the 8–16 cm and 16–32 cm scales resulting from the similar overall colony surface structures of branching and massive *Porites* at these intermediate scales.

Soft coral habitat structural complexity was unexpectedly high, particularly at the smallest scales (4–8 cm), and surprising given that the study method likely underestimates structural complexity of less rigid biota such as soft coral. While the relative contribution of soft corals to reef structural complexity are apparent due to their physical presence when alive, quantification of their structural complexity is complex due to their only partially calcified structures, and has received little attention (though see refs 52, 53). Despite this, structural complexity of these soft coral habitats was found at two distinct scales of measurement (4–8 cm and 16–64 cm), as the two smallest wheels were able to fit in between adjoining colonies often reaching the substratum below, whereas the larger wheels rolled over the surface of colonies suggesting less relief at larger scales. Similarly, there was little medium- to large-scale structure (16–64 cm) in *Pocillopora* habitat due to the small colony sizes and limited space between them, resulting in the larger wheels remaining on the reef surface. Building upon empirical and modelling studies of Caribbean reefs^16, 17, 48^, our findings show that not only is the identity of constituent corals an important driver of habitat structural complexity, but this occurs across scales. Moreover, the size and number of scales of measurement at which structure is available varies substantially among habitats.

Previous work has shown that broad-scale habitat structural complexity, determined by coral composition and reef condition, can drive the taxonomic and functional diversity of reef fish assemblages^22, 54–56^. More specifically however, the range of scales at which habitat structure is available likely regulates how species organisation is partitioned and ecological processes are maintained^1, 3^. Evidence suggests that ecosystems are strongly influenced by processes operating over different scales, and their resilience is determined by diverse, though overlapping, functions at and across those scales^2, 50^. For example, herbivory, a critical process on coral reefs, is mutually reinforced when reef fishes with shared functions can operate across multiple spatial scales, thereby minimising competition between fishes of similar body-sizes^10^. Therefore, a loss of habitat structural complexity at specific scales may compromise resilience^3^, even where habitat-building organisms remain present and appear intact, but have undergone species shifts^57^.

The homogenisation of habitats characterized by increasingly monospecific assemblages of habitat-building species therefore has broader implications than simply the habitat structural complexity of ecosystems. Conservation practitioners responsible for maintaining coral reef ecosystem services are therefore advised to consider changes in the composition of coral assemblages, and not simply total coral cover on reefs^17^. Studies suggest that total coral cover alone is a poor surrogate for habitat structural complexity^17^, the organization of reef associated species^58^, ecological function^59^, or reef recovery^60^, as it does not capture sufficient variation in structural complexity driven by benthic composition. Some coral habitats might warrant relatively greater protection as their inherent variation in habitat structural complexity may support enhanced ecosystem resilience (e.g. *Acropora* and *Orbicella* reefs in the Caribbean, refs 36, 61, 62). The strong linear relationships between structural complexity and the dimension of individual colonies of massive *Porites, P. cylindrica*, and *P. damicornis* suggest that data on the composition of habitat-building species may prove to be a useful proxy for cross-scale habitat structural complexity. In this way, a refined surrogate for habitat structural complexity that combines coral cover and composition may offer a more effective resilience indicator, thereby improving the likelihood of success of important and costly conservation initiatives^61, 63^. Mechanistic models of structural complexity have been developed to describe the structure of Caribbean reefs at a broad scale in relation to shifts in benthic communities, using simplified colony shapes of explicit volumes^48^, or finer scale estimates of coral structural complexity standardised by colony size^36^. Similar models could be developed for Indo-Pacific reefs using emerging low-cost, effective techniques such as photogrammetry^34, 64^, allowing predictions of cross-scale structural dynamics resulting from shifts in dominance patterns of corals in the region.

The likely outcomes of continued coral loss on the structural complexity of coral reef habitats will be largely dependent on the nature, frequency and severity of future disturbances, and the capacity for different coral taxa to adapt to changing conditions^26, 39^. Among those habitats considered here, massive *Porites* generally provided the most structurally complex habitat at each scale, arguably due to the sheer size of colonies. Massive *Porites* are relatively slow-growing and tolerant to stressors such as warm-water anomalies^18^, poor water quality^65^, and large storms^30^, and as such is among those taxa predicted to persist into the future^18^. Similarly, branching *Porites* was structurally complex across scales, but is relatively fast-growing^18^, and exhibits varied levels of sensitivity to thermal stress^66^. This may afford some optimism for future reefs despite escalating anthropogenic disturbance, as persistent corals that can offer refugia across a range of scales have the potential to mediate predator-prey interactions^67^, thereby extending fish body-size distributions^8^, and coral reef food chains^58^. Conversely, the cross-scale habitat structural complexity of degraded reefs with little remaining coral cover (<10%) highlight both the vulnerability of fine scale structure to disturbance, and the more robust nature of larger scale reef structure. The loss of fine scale structure has important implications for species and life stages of fishes that rely on it for refugia, and is likely to lead to rapid reductions in small bodied fish species, and lagged declines in larger bodied species that rely on fine scale habitat structural complexity as juveniles^41, 68^.

Our results provide new insight into the cross-scale structural dynamics of taxonomically distinct coral reef habitats across spatial scales of measurement relevant to refuge selection by fishes^8, 69^. However, the outcomes of assemblage shifts can be diverse and spatially variable, such that quantification of the cross-scale habitat structural complexity of additional coral configurations are warranted. For example, tabular and branching *Acropora* is structurally distinctive and typically dominates large areas of undisturbed coral reef habitats in the Indo-Pacific^60^, but was not locally abundant at Lizard Island during this study. Furthermore, this study focused on shallow, sheltered reefs only, providing scope for broader investigation. Coral species composition and the morphology of some coral species vary with abiotic conditions (e.g. exposure, depth, water flow, light), biological processes (e.g. recruitment, competition, predation), and disturbance histories^39, 70^, likely causing variation in the structural complexity of habitats. Similarly, the structural complexity of degraded coral reef habitats can be highly variable, influenced by local disturbance histories (e.g. coral bleaching versus large storms^71^), the underlying substrate^8^, and the colonisation of other benthic organisms (e.g. macroalgae^51^). Finally, the method employed in this study while useful for capturing some aspects of structural complexity (e.g. spaces under overhangs and in non-vertical recesses) that may be underestimated using approaches such as profile gauges and photographic methods^72^, only captures an estimate of the two-dimensional structural complexity of habitats. Coral reef structures are often multidimensional, with different sized holes and passages throughout the matrix itself. Therefore, in seeking to understand how reef structure relates to the distribution of associated organisms, it would be prudent to consider the specific method used for assessing variation in habitat structural complexity^73^.

Our results provide evidence that habitat structural complexity can be multifaceted over ecologically relevant scales, and demonstrates the importance of going beyond a consideration of just the presence of habitat-building organisms, to include taxonomic structure in efforts to maintain ecosystems and the provision of associated goods and services^37, 58^. Coral reefs are among the world’s most biodiverse but threatened ecosystems^26^. As global conservation increases in response to coral reef degradation^74^, assessments of reef condition and the identification of priority areas for protection should consider the composition as well as cover of corals and other habitat forming organisms. Moreover, identifying inherent patterns of cross-scale habitat structural complexity typical of likely future configurations of species may prove critical for understanding the ecology and conservation of those coral reef systems.

## Methods

### Study location

This study was conducted in September 2015 on the reefs surrounding Lizard Island, a granitic island in the northern Great Barrier Reef, Australia (14°41′S, 145°27′E). Benthic composition and cross-scale habitat structural complexity were quantified at twelve randomly selected sites on the leeward side of the island. All sites were shallow (<6-m depth) reef edges (>5-m wide) adjacent to sand. All sites were in areas protected from the prevailing south-east swell, with comparable water clarity and flow, light levels, and geomorphology. Adjacent sites were separated by a minimum of 500 m.

Benthic composition was quantified along six replicate 30-m point-intercept transects at each site, recording the substratum directly beneath the transect line at 25-cm intervals (120 points per transect). Transects were positioned parallel to the reef edge at a depth of 2–6-m, with a minimum of 5 m between adjacent transects. Substratum types included hard (scleractinian) corals (identified to genus or species where possible, and growth form noted), soft (alcyonacean) corals, ‘other sessile invertebrates’ (primarily sponges, giant clams, and ascidians), macroalgae, erect crustose coralline algae, dead coral and pavement, rubble and sand.

### Habitat structural complexity

Habitat structural complexity was estimated at five spatial scales of measurement following a step-length geometric series using distance measuring wheels of different diameters (4–64 cm) along four 10-m transects at each site (adapted from^72^, following^8^). The 10-m transects used to quantify structural complexity were positioned within the mid-section (i.e., ~10–20 m) of four of the six 30-m transects used to quantify benthic composition. Adjacent 10-m transects were separated by a minimum of 20 m. The abundance of fishes has been shown to positively correlate with structural complexity relative to fish body-size^41, 68^, and the aperture diameter of available holes or crevices in the substrate as refuges from predation or environmental stressors^69^. Therefore, scales of measurement were selected to correspond to the body depths of non-cryptic fish species. The contour distance travelled by each wheel over the reef substratum was estimated by rolling the wheels along the reef surface contour immediately below the length of the taught 10-m transect line, being careful to ensure each wheel followed the detailed surface structure of the benthos (Fig. 1). The number of complete rotations and the proportion of each wheel turned for any incomplete rotations were recorded. The contour distance covered by each wheel was calculated by multiplying the number of rotations by the wheel circumference.

### Colony level structural complexity

To assess how the five scales of structural complexity relate to colony size of corals, we quantified the structural complexity of three of the most common hard coral taxa at the study sites. Contour distance travelled by each wheel was estimated across the maximum diameter of individual colonies of *Porites cylindrica*, massive *Porites* (mostly *Porites lutea*), and *Pocillopora damicornis* (measured to the nearest cm with a tape *in situ* over the surface of the colony). Structural complexity estimates were acquired across the range of available colony sizes for each taxa (*P. cylindrica:* 3–350 cm, *n* = 100; massive *Porites:* 3–415 cm, *n* = 100; *P. damicornis:* 3–69 cm, *n* = 72), at other sheltered reef edge sites around Lizard Island.

### Data analyses

Variation in benthic composition among sites was investigated with non-metric multi-dimensional scaling (nMDS) based on Bray-Curtis similarities of square root transformed benthic cover data in Primer v6^75^. Group-average hierarchical clustering was used to provide an objective assessment of five distinct habitat groups identified with nMDS. Two-way permutational multivariate analysis of variance (PERMANOVA) was used to test the significance of these groupings (9999 permutations), with habitat (fixed; branching *Porites*, massive *Porites*, *Pocillopora*, soft coral, and degraded) and site (random) as factors (PERMANOVA+ add on package). One-way pairwise comparisons between habitat groups were performed with unrestricted permutation of raw data to allow for sufficient permutations to be tested. Similarity Percentage (SIMPER) analysis was used *post hoc* to identify those benthic categories contributing consistently to average similarity within, and dissimilarity between, habitats with a similarity/dissimilarity test ratio of ≥4.0 or 2.0, respectively^75^.

Differences in (i) contour distances measured at each scale were compared among habitats (fixed effect), and (ii) differences in contour distances measured were compared across scales (fixed effect) within each habitat, using linear mixed effects models, with lme in *nlme* in all instances (R version 3.2.3; R Development Core Team 2015). In each analysis, site was treated as a random effect and Tukey multiple comparison tests were used to identify where differences occurred (with the *multcomp* package). Exploratory graphical analysis of model residuals suggested the data conformed to the assumptions of normality and independence, though there was heterogeneity of variance among habitats at the largest scale. Therefore models were fitted with a constant variance structure to allow for heteroscedasticity at all scales, consequently allowing for cross-scale comparisons. To identify the main sources of variation at each scale, variance components were subsequently extracted using *lme4* and the *MuMIn* package (see Supplementary Table S1).

Multiple linear regression was used to estimate relationships between habitat structural complexity with total coral cover (hard and soft coral) and benthic composition (habitat classification) at each scale. Collinearity between coral cover and habitat type was tested by calculating generalized variance inflation-factors (GVIF^1/2*df* ^76^). As GVIF values indicated low levels of collinearity (<3^77^), information-theoretic model selection was used to determine the relative importance of these covariates in predicting variation in habitat structural complexity (*MuMIn* package). Multi-model inference (including null models) was estimated by ranked changes in AICc <3^78^. To determine the scales where changes in structural complexity occurred within habitats, hierarchical modelling was also used to compare contour distances across scales within each habitat, accounting for site effects, followed by Tukey tests. Due to unequal variance across scales within habitats, models were fitted to allow for heterogeneity as previously described. Only one site was identified to be dominated by *Pocillopora*, and subsequently contour distance was compared across scales without site effects for this habitat using the gls function of *nlme*. The relationships between colony size and structural complexity at the same five scales were assessed for massive *Porites*, *P. cylindrica* and *P. damicornis*, using linear regression.

### Data availability

The datasets generated and analysed during the current study are available in the James Cook University Tropical Data Hub repository, https://research.jcu.edu.au/researchdata.

## Electronic supplementary material

Supplementary Information
