# Supplementary material for: Cross-scale habitat structure driven by coral species composition on tropical reefs
Source: Sci Rep. 2017 Aug 8;7:7557. doi: 10.1038/s41598-017-08109-4 (PMC5548803; doi:10.1038/s41598-017-08109-4)
Supplement: Supplementary file 1 — Supplementary Information [file 41598_2017_8109_MOESM1_ESM.pdf]

Cross-scale habitat structure driven by coral species composition on tropical reefs

Laura E. Richardson[1] * laura.richardson1@my.jcu.edu.au; tel. +61 7 4781 6024, Nicholas A.J. Graham [1, 2] nick.graham@lancaster.ac.uk, Andrew S. Hoey [1] andrew.hoey1@jcu.edu.au

[1] ARC Centre of Excellence for Coral Reef Studies, James Cook University, Townsville, QLD 4811, Australia.

[2] Lancaster Environment Centre, Lancaster University, Lancaster, LA1 4YQ, United Kingdom.

Supplementary Information

Supplementary Table S1 Contour distance across scales at each habitat and components of variation in contour distance at each level: Habitat, Site, and Transect. Variance components are shown as percentages of total variance explained (extracted with *lme4* and *MuMIn* in *R*).

Scale (cm)	Wheel (cm)		Mean ± SE contour distance (m)					Variance components (%)		
	Diameter	Circumference	Branching *Porites*	Degraded	Massive *Porites*	*Pocillopora*	Soft coral	Habitat	Site	Transect
4	3.99	12.53	20.59 ± 0.48	15.17 ± 1.07	20.06 ± 1.01	16.68 ± 0.98	17.81 ± 0.99	36.82	0.00	63.18
8	7.97	25.05	16.48 ± 0.85	13.73 ± 0.77	16.60 ± 0.99	12.62 ± 1.17	15.82 ± 0.85	21.13	0.00	78.87
16	15.95	50.10	14.91 ± 0.79	13.42 ± 0.44	15.08 ± 0.62	11.84 ± 0.42	12.81 ± 0.62	22.96	0.00	77.04
32	31.89	100.20	12.71 ± 0.56	12.18 ± 0.30	13.35 ± 0.39	11.30 ± 0.11	11.52 ± 0.29	21.83	0.00	78.17
64	63.79	200.40	11.39 ± 0.21	11.82 ± 0.18	12.08 ± 0.26	10.49 ± 0.09	11.18 ± 0.11	35.05	5.46	59.49

Supplementary Table S2 One-way PERMANOVA pairwise comparisons of benthic composition among coral habitats. Comparisons based on Bray-Curtis similarities of unrestricted permutation of raw data (> 6835 permutations). Benthic variables consistently contributing to average similarity within (sim/SD > 4; grey shaded boxes), and dissimilarity between habitats, (unshaded boxes; diss/SD > 2) identified in one-way SIMPER are listed.

	Branching *Porites*	Massive *Porites*	*Pocillopora*	Soft coral	Degraded
Branching *Porites*	Branching *Porites*<br>Dead coral<br>**Av. sim. 74.7%**	Branching *Porites*<br>Massive *Porites*<br>**Av. dissim. 38.2%**	Branching *Porites*<br>*Pocillopora*<br>**Av. dissim. 50.5%**	Branching *Porites*<br>Soft coral<br>**Av. dissim. 46.2%**	Branching *Porites*<br>Macroalgae<br>**Av. dissim. 44.1%**
Massive *Porites*	**P=0.0001**	Massive *Porites*<br>Dead coral<br>Soft coral<br>**Av. sim. 79.7%**	Massive *Porites*<br>*Pocillopora*<br>**Av. dissim. 42.9%**	Massive *Porites*<br>Soft coral<br>**Av. dissim. 36.3%**	Massive *Porites*<br>Macroalgae<br>**Av. dissim. 35.3%**
Pocillopora	**P=0.0001**	**P=0.0001**	*Pocillopora*<br>Dead coral<br>Soft coral<br>**Av. sim. 80.1%**	Soft coral<br>*Pocillopora*<br>**Av. dissim. 43.4%**	*Pocillopora*<br>Macroalgae<br>**Av. dissim. 45.7%**
Soft coral	**P=0.0001**	**P=0.0001**	**P=0.0001**	Soft coral<br>Dead coral<br>Rubble<br>**Av. sim. 80.9%**	Soft coral<br>Macroalgae<br>**Av. dissim. 44.3%**
Degraded	**P=0.0001**	**P=0.0001**	**P=0.0001**	**P=0.0001**	Dead coral<br>**Av. sim. 76.8%**

Supplementary Table S3 Summary of mixed effects linear regression analyses of habitat (fixed), and site (random) predicting contour distance measured at multiple scales (4-64 cm), and Tukey multiple comparison tests.

Scale (cm)	$F_{4,7}$	P	R^2 (habitat)	Tukey P (all)	Inter-habitat variation
4	6.73	**0.02**	0.64	**≤ 0.01**	Branching *Porites*, Massive *Porites* > Degraded; Branching *Porites* > *Pocillopora*
8	3.32	0.08	0.19	NA	NA
16	6.18	**0.02**	0.14	**≤ 0.01**	Branching *Porites*, Massive *Porites* > *Pocillopora*
32	8.81	**0.01**	0.11	**≤ 0.04**	Degraded, Massive *Porites* > *Pocillopora*; Massive *Porites* > Soft coral
64	4.34	**0.04**	0.25	**< 0.01**	Degraded, Massive *Porites* > *Pocillopora*

Supplementary Table S4 Summary of mixed effects linear regression analyses of contour distance measured at multiple scales (4-64 cm; fixed) within habitats, using site as a random factor, and Tukey multiple comparison tests.

Habitat	F	df	P	R^2 (scale)	Tukey P (all)	Inter-scale (cm) variation
Branching *Porites*	82.92	4,53	**<0.001**	0.79	**<0.001**	4 > 8, 16, 32, 64; 16 > 32, 64; 16 > 64
Degraded	6.27	4,53	**<0.001**	0.09	**< 0.02**	4, 16 > 64
Massive *Porites*	21.37	4,34	**<0.001**	0.49	**< 0.05**	4 > 16, 32, 64; 16 > 32, 64; 16 > 64; 32 > 64
Pocillopora	18.30	4,15	**<0.001**	0.48	**< 0.02**	4 > 8, 16, 32, 64; 16, 32 > 64
Soft coral	20.55	4,43	**<0.001**	0.42	**< 0.04**	4 > 16, 32, 64; 8 > 16, 32, 64

Supplementary Table S5 Regression coefficients and R^2 of contour distance and colony size for *Porites cylindrica*, massive *Porites* (mostly *P. lutea*) and *Pocillopora damicornis.*

Scale (cm)	*P. cylindrica*			Massive *Porites*			*P. damicornis*		
	Intercept	Slope	R^2	Intercept	Slope	R^2	Intercept	Slope	R^2
4	-14.6890	2.2142	0.93	1.52052	1.61221	0.96	-4.037	1.559	0.68
8	-0.27129	1.52378	0.95	3.96408	1.47673	0.95	-2.03474	1.43967	0.78
16	-0.06219	1.34541	0.96	3.09503	1.45661	0.95	-1.91172	1.27025	0.87
32	-0.92919	1.25057	0.97	-0.92543	1.44113	0.97	-2.34713	1.23802	0.90
64	-0.32606	1.18825	0.97	-4.43976	1.40474	0.97	-2.67417	1.19909	0.91
